# Unraveling the Transmission Dynamics of a Novel Norovirus GII.17[P17] Lineage During Two Consecutive Outbreaks in a Spanish Hospital

**DOI:** 10.1002/jmv.70966

**Published:** 2026-05-11

**Authors:** Jasmin S. Kutter, Oscar Cuevas‐Lobato, Blanca Esperanza Fernandez‐Pacheco‐Gonzalez‐Echavarri, Carolina Moreno‐Gomila, Nerea Garcia‐Ibañez, Juan Camacho, Estrella Ruiz‐Pedro, Maria Cabrerizo, Juan‐Ignacio Alós, Francisco Diez‐Fuertes, Maria Dolores Fernandez‐Garcia

**Affiliations:** ^1^ Enterovirus and Viral Gastroenteritis Unit, National Centre for Microbiology Instituto de Salud Carlos III Madrid Spain; ^2^ ECDC fellowship Programme, Public Health Microbiology path (EUPHEM) European Centre for Disease Prevention and Control (ECDC) Stockholm Sweden; ^3^ Servicio de Microbiología Hospital Universitario de Getafe, Getafe Madrid Spain; ^4^ Servicio de Medicina Preventiva Hospital Universitario de Getafe, Getafe Madrid Spain; ^5^ Centro de Investigación Biomédica en Red (CIBER) Epidemiology and Public Health (CIBERESP) Madrid Spain; ^6^ AIDS Immunopathogenesis Unit, National Centre for Microbiology Instituto de Salud Carlos III Madrid Spain; ^7^ Centro de Investigación Biomédica en Red (CIBER) Infectious Diseases (CIBERINFEC) Madrid Spain

**Keywords:** genomic epidemiology, hospital‐associated outbreak, metagenomic next‐generation sequencing, Norovirus, transmission tree

## Abstract

Norovirus outbreaks in healthcare settings pose significant challenges to infection prevention and control (IPC). To prevent and control such outbreaks efficiently, identifying sources and transmission clusters (TCs) is crucial but often limited by traditional outbreak investigations. Here, we examined two consecutive hospital norovirus outbreaks employing a genomic epidemiology approach to elucidate transmission dynamics and guide IPC strategies. Stool samples of 54 symptomatic patients were analyzed with different diagnostic methods, and 26 norovirus‐positive samples underwent metagenomic next‐generation sequencing (mNGS) for phylodynamic and phylogenetic analyses. All infections belonged to the novel GII.17[P17] lineage, circulating globally since 2023/2024. LiquidArray® outperformed fluorescence immunoassay (FIA, 28.6%) and RT‐PCR (85.7%) with FIA's low sensitivity leading to missed cases highlighting the need for molecular confirmation for accurate outbreak management. Genomic analysis revealed multiple introductions, with two TCs identified in Outbreak‐1 and one in Outbreak‐2, as well as inter‐hospital‐unit spread. Reconstruction of transmission trees indicated sustained person‐to‐person spread with 0–3 unobserved intermediate cases in both outbreaks. Identical sequences in patients without clear epidemiological links suggested possible fomite transmission. These analyses provided key insights into infection sources and TCs that would have remained unknown using epidemiological investigations alone, supporting more targeted IPC resource allocation and intervention strategies.

## 1 Introduction

Norovirus is the leading cause of acute gastroenteritis (AGE) across all age groups, affecting 685 million people worldwide annually [1, 2]. It is a highly contagious pathogen capable of causing large outbreaks that are difficult to control. Hospital‐associated outbreaks are of particular concern, imposing considerable risks to patients, especially immunocompromised individuals and the elderly, as well as occupational health, and are associated with significant economic implications [3, 4, 5].

Genotypes GI and GII are the most prevalent cause of human norovirus infections worldwide, with GII.4 being the dominant strain circulating globally [1, 6]. Since the 2023/2024 season, however, a shift from GII.4 to a novel GII.17 lineage has been observed in multiple countries around the world [7, 8], including Spain (unpublished data).

Norovirus genotyping is generally performed using dual polymerase‐capsid PCR [7]. This approach has been effectively used in nosocomial outbreak studies [8, 9, 10]. However, differentiating between sporadic cases, community introductions, and ongoing in‐hospital transmission can be challenging. mNGS offers significantly higher resolution as mutations outside these regions provide valuable phylogenetic information. Together with epidemiological data, the use of mNGS strengthens the identification of well‐supported transmission clusters within outbreaks, providing critical information for IPC measures [11, 12, 13, 14].

In this study, we investigated two consecutive norovirus outbreaks caused by the novel GII.17[P17] lineage that occurred in February and May 2024, affecting elderly patients and healthcare workers (HCWs) in a large hospital. Our aim was to elucidate the transmission dynamics of this novel lineage in both outbreaks, including whether the two outbreaks were related or represented separate introductions, by applying mNGS and integrating genomic data with clinical and epidemiological information. In addition, we conducted a comparative assessment of different diagnostic methods for detecting the novel lineage in stool samples.

## 2 Methods

### 2.1 Setting

The Getafe University Hospital is a secondary‐care hospital with 389‐operational beds (as of 2023/24) located in the municipality of Getafe, Community of Madrid. The hospital has approximately 1900 professionals and an emergency department where norovirus infection cases can be admitted. Oncohematology and Geriatrics are located on the 3rd and 4th floor, respectively. Each unit consists of 23 double‐occupancy rooms distributed across three corridors (Supporting Figure 1). One corridor includes a family waiting room, but there is no common area for patient interaction. A fourth corridor is designated for staff and hospital‐related purposes.

### 2.2 Case Definition

Cases among patients were defined as any patient with AGE symptoms (diarrhea, vomiting, fever) admitted to one of the affected hospital units. Stool samples of symptomatic patients were collected and tested for enteric pathogens at the Microbiology Unit of the hospital. Norovirus testing was performed using a fluorescence immunoassay (FIA, Norovirus Ag FIA, SD BIOSENSOR).

Cases among HCWs were defined as HCWs with self‐reported, gastrointestinal symptoms who worked in one of the affected hospital units. Since testing HCWs is not standard practice, no stool samples from HCWs were available. HCW cases were based on self‐reported gastrointestinal symptoms in affected units, but were not tested, so they were excluded from further analysis.

### 2.3 Outbreaks

We defined an outbreak as ≥2 patients or HCWs with AGE symptoms occurring in a unit exceeding the expected baseline incidence within a certain period. If multiple units were affected simultaneously, the outbreaks were considered a single outbreak. An outbreak was considered resolved, when IPC measures were lifted 48 h after the last reported case.

### 2.4 Description of Events

On February 8, the Preventive Medicine Unit was notified of several AGE cases in Geriatrics. The first case had been identified on January 21, followed by additional AGE cases among patients and HCWs. However, a link between the cases was not identified initially, leading to an 18‐day delay in notification to the Preventive Medicine Unit, which declared an outbreak on February 8. Stool samples were collected from symptomatic patients, and IPC measures were implemented (Supporting Information). Norovirus was detected by FIA in stool samples of five patients (Supporting Table 1). The outbreak was declared resolved on February 15, 48 h after the last reported symptomatic case. At that time, the Preventive Medicine Department had associated a total of 21 patients and 24 HCWs with the outbreak. Shortly thereafter, nine AGE cases were identified in Oncohematology between February 17 and 28. However, no enteric pathogen was detected in stool samples. Consequently, no outbreak was officially declared in this unit, although IPC measures were implemented to prevent transmission. In addition, six symptomatic patients were identified in Geriatrics beyond February 15, the date of outbreak resolution. Two of the collected stool samples from these patients tested positive for *Clostridium difficile (C. difficile)* toxin (Supporting Table 1). Due to the rise in symptomatic cases across both units, on March 7, all available stool samples (38 samples from 32 patients) were sent to the viral gastroenteritis reference laboratory of the National Centre for Microbiology (CNM) for detailed molecular investigation of the etiological agent. For several patients stool samples were not available for analysis, particularly for those who were only associated with the outbreak retrospectively.

In the week of May 7, another AGE outbreak was reported in Geriatrics, this time affecting 12 patients and four HCWs. Norovirus was detected in the stool samples of five, while *C. difficile* was detected in three cases. The outbreak was officially resolved by May 9. Following the additional collection of stool samples from 10 symptomatic patients after the official outbreak resolution, a total of 28 stool samples from 22 symptomatic patients were sent to the CNM to extend the analysis to the second outbreak.

### 2.5 Epidemiological and Clinical Investigations

A descriptive analysis was performed on patient demographics and clinical characteristics (Table 1, Supporting Tables 2–4). Floor plans of Geriatrics and Oncohematology (Supporting Figure 1) were used to allocate patients to rooms and analyze potential contacts and transmission chains.

**Table 1:** Demographic and clinical characteristics of symptomatic patients whose stool samples were analyzed by outbreak and hospital unit.

	Total cases *N* = 54	Total outbreak 1 *N* = 32	Outbreak 1		Outbreak 2
Characteristic			Oncohematology *N* = 9	Geriatrics *N* = 23	Geriatrics *N* = 22
**Age**					
Median	86	86	85	88	87
Range	47–96	47–95	47–89	71–95	67–96
**Gender, *n* (%)**					
Female	39 (72.2)	24 (75.0)	7 (77.8)	17 (73.9)	15 (68.2)
Male	15 (27.8)	8 (25.0)	2 (22.2)	6 (26.1)	7 (31.8)
**Hospitalization period (d)**					
Median	13	12	18	11	13
Range	3–56	4–45	5–40	4–45	3–56
**Symptoms *n* (%)**					
Diarrhea	45 (84.9)	32 (100)	9 (100)	23 (100)	13 (61.9)
Fever (>38°C)	13 (24.1)	11 (34.4)	2 (22.2)	9 (39.1)	2 (9.1)
Vomiting	10 (18.5)	9 (28.1)	1 (11.1)	8 (34.8)	1 (4.5)
Asymptomatic	6 (11.3)	0 (0.0)	0 (0.0)	0 (0.0)	6 (27.3)
**Symptom duration (hrs.)**					
Mean (SD)	45 (20.0)	44 (23.1)	35 (23.9)	48 (23.1)	48 (12.8)
**Discharge status, *n* (%)**					
Alive	45 (83.3)	27 (84.4)	8 (88.9)	19 (82.6)	18 (81.8)
Dead*	9 (16.7)	5 (15.6)	1 (11.1)	4 (17.4)	4 (18.2)
**Norovirus positive, *n* (%)**					
**FIA**					
Yes	10 (18.5)	5 (15.6)	0 (0.0)	5 (21.7)	5 (22.7)
No	44 (82.5)	27 (84.4)	9 (100)	18 (78.3)	17 (77.3)
**RT‐PCR** ‡					
Yes	26 (48.1)	17 (53.1)	3 (33.3)	14 (60.9)	9 (40.9)
No	28 (51.9)	15 (46.9)	6 (66.7)	9 (39.1)	13 (59.1)
**Liquid Array®**					
Yes	30 (55.6)	19 (59.4)	5 (55.6)	14 (60.9)	11 (50.0)
No	23 (42.6)	13 (40.6)	4 (44.4)	9 (39.1)	10 (45.5)¥

* Norovirus was not the cause of death.

‡ Confirmed by Sanger sequencing.

¥ One sample was invalid.

### 2.6 Norovirus Detection and Genotyping

Dual polymerase‐capsid genotyping was performed by conventional RT‐PCR protocol for GII followed by Sanger sequencing [7] (Supporting Information). Detection of norovirus in stool samples was further performed using the LiquidArray® Gastrointestinal VER 1.0 (Bruker UK/Hain Lifescience GmbH) according to the manufacturer's protocol [15] (Supporting Information). The detection rate of FIA and RT‐PCR relative to LiquidArray® was calculated using the total number of stool samples (*n* = 66).

### 2.7 mNGs and Phylogenetic Analysis

Hybrid capture‐based mNGS was performed as described previously [16]. Consensus sequences were generated by mapping reads to a reference sequence (Supporting Information). Evolutionary rates were estimated in BEAST v.1.10.4 using the General Time Reversible model with gamma rate distribution and invariable sites parameter with 100 million Markov chain Monte Carlo (MCMC) runs to reach convergence of all parameters; all evolutionary rates were calculated using the strict clock model and coalescent constant size tree prior [17, 18] (Supporting Information). Several whole‐genome sequences closely related to those of Outbreaks‐1 and ‐2 and collected in 2023/2024 were identified through BLAST searches and included in phylogenetic analyses. These sequences were used to estimate the evolutionary rate of the recently circulating GII.17[P17] strains and the maximum clade credibility (MCC) trees. Phylogenetic trees of patient sequences were constructed using the maximum likelihood (ML) method using the Tamura‐Nei (TN93) model [19] with invariable sites parameter and 1000 bootstrap replicates. Pairwise SNV distances were analyzed at consensus‐level. Both analyses were performed using MEGA 11 software (Version 11.0.13.).

### 2.8 Transmission Clusters

Transmission clusters were defined as ≥2 sequences sharing a common ancestor and had a node support >95%.

### 2.9 Transmission Tree Reconstruction

Transmission trees were reconstructed using the *outbreaker2* model [20, 21, 22]. The evolutionary rate from this study was used as the mutation rate. Generation time (mean: 1.86 days [23], SD: 1.21) and incubation period (mean 1.40 [24] days, SD: 0.23 days) were described by discretized gamma distributions (Supporting Information). For each outbreak, four MCMC chains with random seed were run with 10^6^ iterations, a thinning frequency of 1/1000, and a burn‐in of 10%. The consensus trees were subsequently visualized with the R package igraph [25].

## 3 Results

### 3.1 Both Outbreaks Were Caused by Norovirus GII.17[P17]

**Figure 1:**
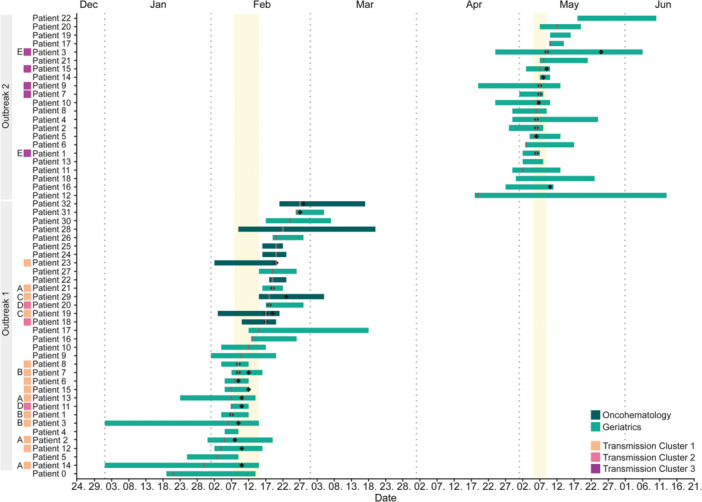
Gantt chart illustrating the hospitalization period of each patient. Vertical gray lines represent the date of symptom onset. Black diamonds indicate the collection date of a norovirus‐positive stool sample. Light yellow rectangles indicate the period between the official outbreak declaration and resolution by the infection prevention department. Letters A–E indicate patients with identical sequences.

For both outbreaks, the median time between date of symptom onset and sample collection was 1 day (IQR: 0–3 days, Figure 1). The late identification of Outbreak‐1 resulted in a longer delay to sample collection among patients with symptom onset before the outbreak declaration with a median of 3 days (IQR:3–5 days, Figure 1). In total, 66 stool samples of 54 patients were processed (Supporting Table 1). RT‐PCR identified 30 (45.5%, 30/66) GII.17[P17]‐positive stool samples, corresponding to 26 (48.1%, 26/54) symptomatic patients: Seventeen (53.1%, 17/32) patients in Outbreak‐1, including three (33%, 3/9) patients from Oncohematology, and nine (40.9%, 9/22) in Outbreak‐2 (Table 1, Supporting Table 1). Using the LiquidArray® four additional norovirus GII‐infected patients from Outbreak‐1 and ‐2 could be identified, confirming a norovirus infection in 30 (55.6%, 30/54) patients across both outbreaks (Table 1, Supporting Table 1). Stool samples from eight patients in Outbreak‐1 and two patients in Outbreak‐2 tested positive for norovirus after the respective outbreak resolution dates (Figure 1). The relative detection rate of FIA and RT‐PCR compared to LiquidArray® was 28.6% (10/35) and 85.7% (30/35).

### 3.2 Genomic Analysis

Near‐complete genomes (99.9% average coverage) could be generated from 24/26 (92.3%) stool samples of 22 norovirus‐positive patients: 18 from Outbreak‐1 and 6 from Outbreak‐2 (Supporting Table 1). From Patient 19 (Oncohematology, Outbreak‐1) and Patient 3 (Geriatrics, Outbreak‐2), a second norovirus‐positive stool sample was obtained 2 and 16 days after the collection of the first stool sample, respectively, and included in the whole‐genome analysis. mNGS confirmed the genotype GII.17[P17] in all cases, and mNGS‐generated consensus sequences were identical to Sanger sequences.

**Figure 2:**
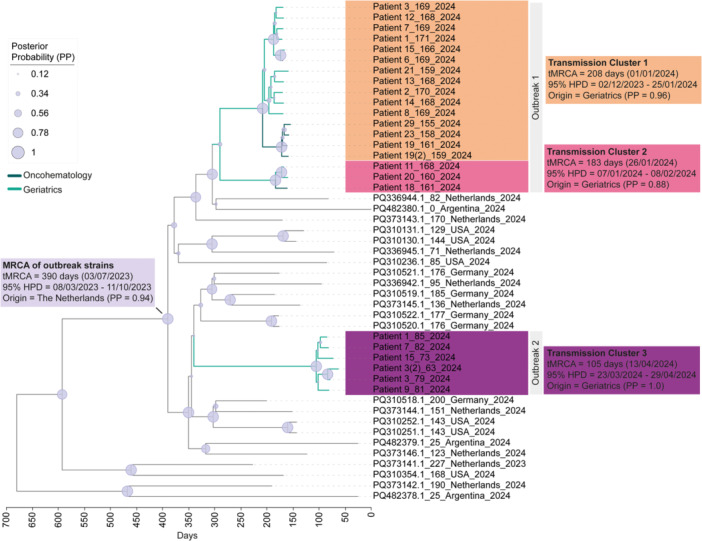
Bayesian MCC tree of the 24 patient‐derived whole‐genome sequences together with closely related genomes from GenBank. Patient sequence labels include patient ID, days to the most recent common ancestor (MRCA), and year of isolation. GenBank sequence labels indicate the accession number, days to MRCA, country of origin, and year of isolation. Node size reflects posterior probability (PP) values. The scale axis indicates the estimated dating of the MRCA. Patient genome sequences are available under accession numbers PV750875‐PV750898. HPD, highest posterior density; MCC, maximum clade credibility; tMRCA, time to most recent common ancestor.

Phylodynamic analysis revealed three distinct transmission clusters (TC) 1–3 (Figure 2). Sequences of Outbreak‐1 branched into TC1 and TC2, with the most recent common ancestor (MRCA) emerging in early and late January 2024, respectively. Both clusters included sequences from patients hospitalized in Oncohematology, phylogenetically linking cases across the two units. For both clusters, Geriatrics was the most probable origin (*PP* = 0.96; *PP* = 0.88). Outbreak‐2 branched into TC3 independent of Outbreak‐1 with the MCRA emerging mid‐April 2024. The MRCA of all three TCs presumably emerged in the Netherlands (*PP* = 0.94) in 2023 (Figure 2).

The mean evolutionary rate was 3.696 × 10^−3^ substitutions/site/year (SEM: 3.28 × 10^−3^), corresponding to 0.53 SNV/genome/week. Given the 6‐week span between the first and last recorded symptomatic patient of Outbreak‐1, we estimated that sequences grouped within the same TC would be expected to differ by an average pairwise distance of ≤3.2 SNVs. For Outbreak‐2, which spanned 3 weeks, the corresponding threshold was ≤1.6 SNVs.

**Figure 3:**
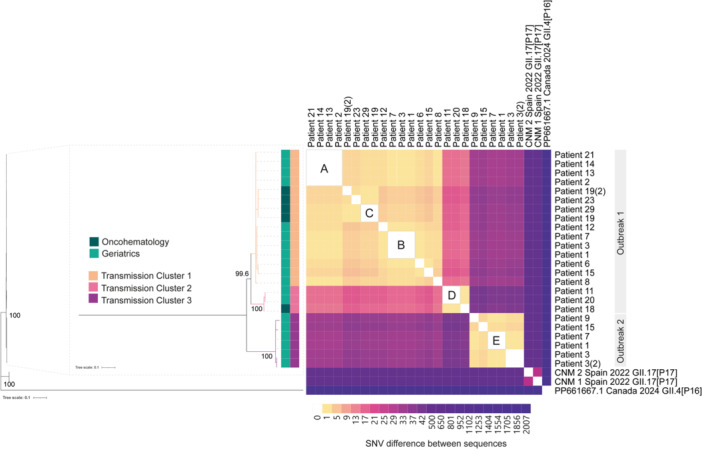
ML tree and heatmap of SNV pairwise distance (number of nucleotides) of 24 patient‐derived whole‐genome sequences. Letters A–E indicate identical sequences (SNV = 0). Two GII.17[P17] strains from Spain (2022) were included as reference (accession numbers: PV750899 and PV750900). A GII.4[P16] strain from Canada (2023, accession number PP661667) served as outgroup. Bootstrap values ≥90 are displayed at the respective nodes.

To validate and further investigate the TCs identified in the phylodynamic analysis, we performed a ML phylogeny and nucleotide pairwise distance analysis, both of which confirmed the results. Sequences branched into three distinct TCs with high bootstrap support (>99, Figure 3). TC1 grouped 14 patients, including three from Oncohematology, while TC2 grouped three patients, two (Patient 11 and 20) from Geriatrics, and one (Patient 18) from Oncohematology. The mean pairwise distance between sequences of TC1 and TC2 was 2.1 SNVs (range 0–6) and 0.4 SNVs (range 0–1), respectively. In Outbreak‐2, all obtained whole genomes were grouped in TC3 with a mean pairwise distance of 1.5 SNVs (range 0–3). SNV analysis also revealed five groups of identical sequences (SNV = 0), three in TC1 (A–C) and one each in TC2 (D) and TC3 (E) (Figure 3).

The mean pairwise distance between TC1 and 2 was 17 SNVs (range 15–19) and between the first two clusters and TC3 was 35.3 SNVs (range 32–42), showing that the clusters are genetically well‐defined.

Comparison with two Spanish GII.17[P17] strains isolated in 2022 revealed a clear genetic distinction to the three clusters with a mean pairwise distance of 524.5 SNVs (range 515–537) (Figure 3).

Interestingly, the sequences obtained from two consecutive samples from Patient 3 (Outbreak‐2), collected 16 days apart, were identical. In contrast, the sequence obtained from the second sample of Patient 19 in Oncohematology (Outbreak‐1), collected only 2 days after the first already harbored one SNV.

### 3.3 Epidemiological Analysis

**Figure 4:**
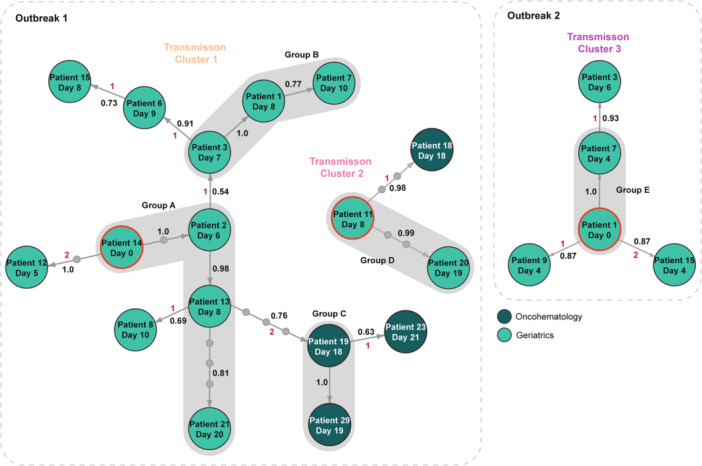
Transmission trees for each Transmission Cluster (TC). Circles are labeled with patient ID and day of symptom onset relative to the earliest case (day 0). Red outlines denote imported cases, i.e., cases with no known infector. Arrows indicate infection direction. Small gray circles on arrows indicate the number of unobserved intermediate cases between patients. Black numbers represent the posterior probabilites (PP) for each transmission chain, while red numbers indicate the number of SNV differences between sequences. Gray rounded rectangles group patients with identical sequences.

Spatiotemporal analysis of patients in both outbreaks revealed that only three patient‐pairs with confirmed norovirus infections were sharing a room (Supporting Figure 1). Among those with available whole genomes, only one pair had identical norovirus sequences (Group E). The remaining patients were either admitted to different rooms and/or corridors, or to the same room after discharge of the previous patient (Figure 1, Supporting Figure 1). Therefore, to investigate potential infection sources and transmission chains, we reconstructed probabilistic transmission trees (i.e., networks of who‐infected‐whom) by integrating genomic, temporal, and contact data. For Outbreak‐1, two separate transmission trees were inferred, consistent with the two genetically identified TCs (Figure 4). Both trees revealed well‐supported transmission chains (PP ≥ 0.75) with 0–3 unobserved intermediate cases between patients. The trees further showed that each TC originated from a single imported case (Patients 14 and 11) that each directly generated 2 secondary infections. From Patient 2, the transmission chain branched in two directions, triggering sustained onward transmission in both. Inference further predicted that transmission to Oncohematology originated from Geriatrics consistent with the phylodynamic results. Specifically, transmission to Oncohematology likely originated from Patients 13 (TC1) and 11 (TC2) through 3 and 2 unobserved intermediate cases, respectively. Additional unobserved intermediate cases were also predicted between Patients 13 and 21 (TC1, Group A) and Patients 11 and 20 (TC2, Group D), who had identical sequences, but no identifiable epidemiological link, as they were not hospitalized at the same time.

The inferred transmission tree of Outbreak‐2 (TC3) also showed well‐supported transmission chains (*PP* ≥ 0.85) with no unobserved intermediate case predicted, suggesting direct transmission between patients (Figure 4). One imported case (Patient 1) was identified who directly caused three secondary cases and a fourth (Patient 3) through Patient 7.

## 4 Discussion

A challenge in studying intra‐hospital transmission of norovirus over a short time frame is the high similarity and close phylogenetic relationship of viral strains. Here, we used phylodynamic and phylogenetic analyses of whole‐genome sequences to demonstrate that two consecutive outbreaks in a hospital originated from separate introductions rather than sustained transmission within the hospital, indicating that the IPC measures implemented during Outbreak‐1 were effective. We also show that Outbreak‐1 was driven by two different introductions, while a single introduction was most likely responsible for norovirus infections during Outbreak‐2. However, in this outbreak, multiple introductions cannot be ruled out with certainty because whole‐genome sequences were obtained from only 45.5% of norovirus‐infected patients.

We further established transmission links between Geriatrics and Oncohematology, which had not been recognized in earlier hospital IPC investigations. These findings underscore the importance of integrating genomic data into epidemiological investigations.

The origin of the initial norovirus introduction triggering TC1 in Geriatrics remains unclear. The first recorded case of AGE symptoms occurred in January (Patient 0, Figure 1), but due to the delayed identification of the outbreak, no sample from this patient was available, leaving uncertainty about whether this patient was indeed infected with norovirus. Delayed outbreak recognition may have resulted from nonspecific symptoms. Many patients received gastrointestinal‐affecting treatments such as antibiotics, supplements, or enteral nutrition. This likely made AGE harder to identify, and epidemiological links between cases more difficult to establish. Although IPC measures restricted HCWs to a single unit, their delayed implementation due to late outbreak recognition may have permitted cross‐unit spread. The earliest confirmed case in TC1, Patient 14, likely triggered sustained onward transmission within Geriatrics and facilitated cross‐transmission to Oncohematology. TC2 likely originated from Patient 11, a patient coming from a residential home visiting the emergency department with AGE symptoms, who was later hospitalized in Geriatrics. This case exemplifies a likely community introduction that led to transmission within and between two units. The outbreak also coincided with a substantial increase in AGE cases among HCWs beyond Geriatrics. Combined with the fact that both introductions lead to onward transmission in a second unit, these events underscore the need for enhanced surveillance and stringent IPC measures. Early identification of new introductions and prompt linking of cases, particularly in vulnerable units as well as emergency and outpatient settings, is crucial for early outbreak control to prevent transmission within and between units. Systematic sampling of HCWs was not standard practice, and no stool samples were collected from symptomatic HCWs. Although the outbreaker2 model allows for probabilistic inference of unobserved intermediate cases, the absence of laboratory confirmation of norovirus infection among HCWs, means that their involvement remains a plausible but unconfirmed route of transmission. Notably, prior to outbreak recognition, HCWs worked across units, representing a potential mechanism for cross‐unit spread, despite the absence of direct patient transfers between the two units during the outbreak. These findings highlight the importance of tailored IPC protocols for HCWs, including systematic testing, and extending such measures to other staff (e.g., cleaning personnel) to improve outbreak control and transmission chain reconstruction.

In both outbreaks, norovirus circulation persisted beyond the official resolution, with one case demonstrating prolonged viral shedding by testing positive in stool samples collected 16 days apart. This finding aligns with previous studies showing that viral excretion can continue for weeks after symptom resolution, particularly in older or immunocompromised patients, potentially facilitating transmission even when symptoms have resolved [1, 26, 27]. Taken together, these observations highlight the need for increased awareness of prolonged infectiousness after symptom resolution, especially in vulnerable populations such as those in Geriatrics and Oncohematology and emphasize the importance of maintaining extended IPC precautions. Repeated testing after symptom resolution may provide valuable guidance for safely discontinuing these measures, particularly in high‐risk healthcare settings. Additionally, our findings suggest that official guidelines recommending termination of IPC measures 48 h after symptom resolution may warrant reconsideration [28, 29].

In both outbreaks, we observed groups of identical sequences, often without strong epidemiological links. Particularly where transmission tree reconstruction could not infer a direct link between these patients, fomite transmission can be a likely explanation. However, in cases with clear epidemiological links, person‐to‐person transmission remains plausible, especially considering the low mutation rate of 0.53 SNV/genome/week. Future investigations should incorporate environmental sampling. This could help confirm infection sources and transmission routes, ultimately supporting the development of more effective IPC strategies and enhanced environmental hygiene.

Rapid norovirus detection in hospitals is crucial for outbreak prevention and control. Despite their low sensitivity, immunochromatographic assays are commonly used in hospitals for norovirus detection due to the cost, equipment needs, and turnaround time of molecular methods, which remain the gold standard [30, 31]. Comparing the detection rates between FIA and LiquidArray® revealed that FIA detected fewer than one‐third of the norovirus‐positive samples identified by LiquidArray®, confirming the findings of previous studies [30, 31]. This had significant consequences for Outbreak‐1, during which no official outbreak was declared in Oncohematology because stool samples from AGE patients tested negative for norovirus by FIA. This clearly demonstrates that while immunochromatographic assays can serve as an initial screening tool, in suspected outbreaks, negative results must be confirmed by molecular methods to accurately identify cases. This also highlights the need for clinicians to be aware of the low sensitivity of these assays to correctly associate patients to outbreaks.

In 2024, several European countries, including Spain (unpublished data), observed an unusual rise in norovirus infections, marked by a shift to genotype GII.17 [32, 33]. The high proportion of GII.17‐positive outbreaks in Spain indicates increased circulation and dominance of this genotype. Comparison of the norovirus outbreak strains from this study with GII.17[P17] strains obtained in Spain in 2022 revealed that the strains were genetically distant, while the outbreak strains were closely related to strains circulating in the Netherlands, Argentina, USA, and Germany in 2023–2024. This aligns with the findings of Chhabra et al. [33], who showed that currently circulating GII.17 strains have considerably mutated from earlier GII.17 strains.

## 5 Conclusion

In summary, by combining different detection methods and genomic epidemiology in a hospital‐associated outbreak, this study improved the understanding of infection sources and transmission chains that epidemiological investigations alone would have missed. This genomic epidemiology approach enabled us to determine whether the second outbreak was a continuation of the first or caused by a new norovirus introduction and whether the outbreaks were driven by ongoing in‐hospital transmission or separate introductions. Our findings provide scientific evidence to help improve and adapt IPC measures, ensuring that future outbreaks can be better controlled and prevented. They also enhance the understanding of norovirus infection sources and transmission dynamics within the hospital. Applying the herein used concepts in real‐time, including minor variant analysis, could further improve the allocation of targeted IPC resources and interventions.

## Author Contributions

**Jasmin S. Kutter:** ethical approval acquisition, epidemiological and microbiological investigation, data curation, data analysis, visualization, writing – original draft and revision. **Oscar Cuevas‐Lobato:** ethical approval acquisition, microbiological investigation, revision. **Blanca Esperanza Fernandez‐Pacheco‐Gonzalez‐Echavarri:** clinical and patient data curation, revision. **Carolina Moreno‐Gomila:** clinical and patient data curation, revision. **Nerea Garcia‐Ibañez:** microbiological investigation. **Juan Camacho:** microbiological investigation. **Maria Cabrerizo:** revision. **Juan‐Ignacio Alós:** revision. **Maria Dolores Fernandez‐Garcia:** funding acquisition, writing – original draft and revision, supervision.

## Disclosure

The author is a fellow of the ECDC Fellowship Programme, supported financially by the European Centre for Disease Prevention and Control (ECDC). The views and opinions expressed herein do not state or reflect those of ECDC. ECDC is not responsible for the data and information collation and analysis and cannot be held liable for conclusions or opinions drawn.

## Ethics Statement

This study was approved by the Ethics Committee for Research with Medicines (CEim) of the Getafe University Hospital (CEIm24/39).

## Conflicts of Interest

The authors declare no conflicts of interest.

## Supporting information

Supporting File

## Data Availability

The data that support the findings of this study are available in the supporting material of this article.
