# Supplementary material for: Unraveling the Transmission Dynamics of a Novel Norovirus GII.17[P17] Lineage During Two Consecutive Outbreaks in a Spanish Hospital
Source: J Med Virol. 2026 May 11;98:e70966. doi: 10.1002/jmv.70966 (PMC13161831; doi:10.1002/jmv.70966)
Supplement: Supplementary file 1 — Supporting File [file JMV-98-e70966-s001.docx]

**Supplementary Information**

*Infection control measures*

All patients with gastroenteritis received enteric contact precautions during the outbreaks. Visits for symptomatic patients on the units were limited.  During the outbreak and for up to 48 hours afterwards, there were no new admissions allowed to the affected units. HCWs were only allowed to work on one unit, to prevent virus transmission to other units. Throughout the outbreaks, there was heightened emphasis on hand hygiene practices, proper glove use, isolation of symptomatic patients, and testing of patients with gastroenteritis. Within the first few hours after outbreak detection, initial communication took place with HCWs on the affected units to obtain information, provide recommendations to reduce transmission, and offer guidance on identifying new cases. Regular meetings were held with department leaders (nursing and medicine) and with hospital managers to make decisions related to the outbreak.
The occupational health department was informed of the situation and attended to cases among HCWs, although samples could not be collected. HCWs were also frequently reminded of the steps to take if they developed gastrointestinal symptoms.
Cleaning services were increased so that environmental surfaces throughout the plant were regularly cleaned with bleach during the outbreak. Environmental cleaning focused on regular disinfection of high-touch surfaces with bleach throughout the facility.

*Stool sample preparation for RT-PCR and WGS*

Stool samples were diluted 1:10 with phosphate-buffered saline (PBS), vortexed for 30s, and clarified by centrifugation at 4,000 × g for 10 min.

*RNA extraction and RT-PCR*

RNA was extracted from clarified faecal suspensions using the QIASymphony system (Qiagen) according to the manufacturer's protocol. The extracted RNA was subsequently used for dual polymerase-capsid genotyping by conventional RT-PCR using the One-Step RT-PCR Kit (Qiagen). PCR products were visualized by gel electrophoresis using a 1x TAE 1% agarose gel. The samples for which a visible band was obtained were purified and sent to the genomics department of the ISCIII for Sanger sequencing. For genotyping, the online Norovirus typing tool was used (<https://www.rivm.nl/mpf/typingtool/norovirus/>).

*Stool sample preparation and LiquidArray^®^ Gastrointestinal VER 1.0*

LiquidArray^®^ Gastrointestinal VER 1.0 (Bruker UK/Hain Lifescience GmbH, Nehren, Germany) is a state-of-the-art multiplexing technology for the detection of 26 bacterial, viral, and parasitic enteropathogens and associated toxins. This assay can differentiate between Norovirus GI and GII by targeting their ORF1-ORF2 junctions and detects all GI and GII genotypes (with possible exception of GII.23 and GII.24). The LiquidArray^®^ technology utilizes an asymmetric PCR to generate excess single-stranded amplicons with Lights-On/Lights-Off probes during which a characteristic fluorescence signature is created ensuring higher sensitivity than traditional detection methods.

Stool samples were stabilized in Stool Buffer VER1.0 before subjecting them to semi-automated nucleic acid extraction using the GXT NA Extraction Kit VER 1.0 and GenoXtract^®^ device. Real-time PCR and/or melting curves were performed using the LiquidArray^®^ Gastrointestinal VER 1.0 PCR Kit and the FluoroCycler^®^ XT thermocycler. Fluorescence signatures were subsequently interpreted by the FluoroSoftware XT-IVD software (Version 1.0.1.5.5.75) and results summarized in the FluoroCycler^®^ Report.

*Library generation and metagenomic whole-genome sequencing*

Sample library preparation was conducted using the NEBNext Ultra II Directional RNA Library Prep Kit for Illumina with NEBNext Multiplex Oligos for Illumina (New England BioLabs Inc., Ipswich, United States (US)). Target enrichment was performed by hybrid capture using the Twist Comprehensive Viral Research Panel version 2 (Twist Biosciences, San Francisco, US). Enriched libraries were sequenced on an Illumina NextSeq500 (300 Cycles). Host reads were removed for analyses. The resulting data were analysed using the viralrecon pipeline (<https://github.com/nf-core/viralrecon>). The resulting raw reads were analysed for quality using FastQC version 0.11.9^1^ and then trimmed using fastp version 0.20.1^2^. SPAdes v3.14.0 in metaSPAdes mode was used to perform a de novo assembly of nonhost reads^3^. Consensus sequences were generated by mapping reads to a reference genome (GenBank accession number MT729791.1) using Bowtie2^4^. Multiple sequence alignment was performed with ClustalW and MAFFT. A maximum likelihood phylogenetic tree was generated using the TN93 model with invariable sites parameter and 1000 bootstraps. Phylogenetic tree editing and heatmap construction from pairwise distances was performed in iTOL.

*Transmission tree reconstruction*

Transmission trees were reconstructed using the *outbreaker2* R package^5,6^ based on dates of symptom onset. The *outbreaker2* model combines genomic, temporal, and contact data in a Bayesian framework. Parameters for generation time and incubation period were retrieved from literature (**Table 1**). Standard deviation (SD) values were estimated from reported mean, 95% confidence intervals (CI), and sample size. Contact data were inferred from spatiotemporal information on patients. For each pair of patients, overlap between hospitalization periods was assessed. Among patients with overlapping hospitalization periods, spatial proximity was used to define plausible contact. A binary contact list was constructed in which contact was considered possible when patients were hospitalized simultaneously and located within at least the same corridor. Patient pairs without temporal overlap or located in different corridors or wards were considered unlikely to have had contact.The resulting binary contact list was used as input for transmission tree reconstruction using the outbreaker2 package. Each MCMC chain was assessed for convergence using trace and autocorrelation plots, effective sample size (ESS) estimates and Gelman-Rubin statistics.

Parameter values used to describe NoV generation time and incubation period in outbreaker2.

|  | **Mean** | **95% CI** | **Sample size** | **Reference** |
| --- | --- | --- | --- | --- |
| **Generation time** | 1.86 days | 1.6 - 2.2 days | 65 | ^7^ |
| **Incubation period** | 33.5 hrs | 31.6 - 35.4 hrs | 34 | ^8^ |

*Supplementary Note 1. Epidemiological characteristics of the patient population*

In Outbreak-1 (n=32) the median age was 86 years (range, 47-95) and 25 patients (78.1%) were female (**Table 1**). All patients experienced diarrhoea, while 11 (34.4%) also had fever and nine (28.1%) had vomiting. Mean symptom duration was 44 hours (SD 23.1). In Outbreak-2 (n=22) the median age of patients was 87 years (range, 67-96) and 15 patients (68.2%) were female. Six patients (11.3%) were asymptomatic, while 13 (61.9%) experienced diarrhoea, two (9.1%) had fever, and one (4.5%) had vomiting. Mean symptom duration was 48 hours (SD 12.8). Reason for hospitalization, treatment regimens, and comorbidities are shown in **Supplementary Table 2-4.** Among all patients (n=54), the most common reason for hospitalization was pneumonia (29.6%), followed by an infection other than pneumonia (11.1%). Consequently, most common drug treatment was antimicrobial therapy (60.0%). In Outbreak-2, three patients (Patient 39, 49, and 51) were hospitalized for AGE symptoms. Most common comorbidity was hypertension (75.9%), followed by dyslipidaemia (44.4%) and diabetes mellitus (33.3%).

*Supplementary Note 2. Detection of pathogens other than NoV among suspected and confirmed NoV cases*

Co-infections with respiratory viruses were identified through WGS in three patients (**Supplementary Table 1**). For Patients 6 and 14 (Outbreak-1) near-complete sequences (99.9% average coverage) for Rhinovirus, and for Patient 3 (Outbreak-2) a near-complete sequence (99.6% average coverage) for Human parainfluenza virus 3 could be recovered. In addition, near-complete genomes of nine Rotavirus A segments (95.1% average coverage) could be recovered from the sample of Patient 22. The Rotavirus infection in this patient could be confirmed with LiquidArray^®^. *C. difficile* toxin infections that were detected in five patients (Outbreak-1: Patient 20 and 27, Outbreak-2: Patient 12, 17, and 18) by the hospital could also be confirmed with LiquidArray^®^. Moreover, we were able to identify one *Campylobacter* spp. (Patient 32, Outbreak-1), one *Salmonella* spp. (Patient 12, Outbreak-2), and two ETEC (lt/st) (Patient 5 and 10, Outbreak-2) infections with LiquidArray^®^, which were not detected in the hospital (**Supplementary Table 1**).

**References**

1. Andrew, S. FastQC: a quality control tool for high throughput sequence data (2010). <http://www.bioinformatics.babraham.ac.uk/projects/fastqc>. Last accessed: 18.06.2025.

2. Chen, S., Zhou, Y., Chen, Y. & Gu, J. fastp: an ultra-fast all-in-one FASTQ preprocessor. *Bioinformatics* **34**, i884–i890 (2018).

3. Nurk, S., Meleshko, D., Korobeynikov, A. & Pevzner, P. A. metaSPAdes: a new versatile metagenomic assembler. *Genome Res* **27**, 824–834 (2017).

4. Langmead, B. & Salzberg, S. L. Fast gapped-read alignment with Bowtie 2. *Nat Methods* **9**, 357–9 (2012).

5. Campbell, F. *et al.* outbreaker2: A modular platform for outbreak reconstruction. *BMC Bioinformatics* **19** (Suppl. 11), 363 (2018).

6. Jombart, T. *et al.* outbreaker2: Bayesian Reconstruction of Disease Outbreaks by Combining Epidemiologic and Genomic Data. *PLoS computational biology* **10**, e1003457 (2014).

7. Heijne, J. C. M. *et al.* Enhanced Hygiene Measures and Norovirus Transmission during an Outbreak. *Emerg Infect Dis* **15**, 24–30 (2009).

8. Harris, J. P., Lopman, B. A., Cooper, B. S. & O’brien, S. J. Does spatial proximity drive norovirus transmission during outbreaks in hospitals? *BMJ open* **3**, e003060 (2013).

**Supplementary Tables and Figures**

**Supplementary Table 1.** Pathogen detection in stool samples of patients with different detection methods.

|  |  |  | **In-hospital analysis** | | | | **Viral gastroenteritis reference laboratory (CNM)** | | | |
| --- | --- | --- | --- | --- | --- | --- | --- | --- | --- | --- |
| **Patient** | **Sample number** | **Collection date** | **Stool culture** | **FIA**  **(NoV)** | **Rota-virus** | **PCR *C. diff* toxin** | **RT-PCR** | **LiquidArray®** | **mNGS** | **mNGS coverage (%)** |
| **Outbreak 1** | | | | | | | | | | |
| **Patient 1** | 1 | 07/02/2024 | neg | pos | neg | neg | NoV GII.17[P17] | NoV GII | NoV GII.17[P17] | 99.9 |
| **Patient 2** | 1 | 08/02/2024 | neg | pos | neg | neg | NoV GII.17[P17] | NoV GII | NoV GII.17[P17] | 100 |
| **Patient 3** | 1 | 08/02/2024 | neg | pos | neg | neg | NoV GII.17[P17] | NoV GII | NoV GII.17[P17] | 99.9 |
| **Patient 4** | 1 | 08/02/2024 | neg | neg | neg | neg | neg | neg | neg | NA |
| **Patient 5** | 1 | 08/02/2024 | neg | neg | neg | neg | neg | neg | NT | NA |
| **Patient 6** | 1 | 09/02/2024 | neg | neg | neg | neg | NoV GII.17[P17] | NoV GII | NoV GII.17[P17]  Human rhinovirus 1 | 99.9  99.7 |
| **Patient 7** | 1 | 09/02/2024 | neg | neg | neg | neg | NoV GII.17[P17] | NoV GII | NoV GII.17[P17] | 1 |
|  | 2 | 12/02/2024 | neg | neg | neg | neg | neg | NoV GII | NT | NA |
| **Patient 8** | 1 | 09/02/2024 | neg | neg | neg | neg | NoV GII.17[P17] | NoV GII | NoV GII.17[P17] | 100 |
| **Patient 9** | 1 | 12/02/2024 | neg | neg | neg | neg | neg | neg | neg | NA |
|  | 2 | 20/03/2024 | neg | neg | neg | neg | neg | neg | neg | NA |
| **Patient 10** | 1 | 12/02/2024 | neg | neg | neg | neg | neg | neg | NT | NA |
| **Patient 11** | 1 | 10/02/2024 | neg | pos | neg | neg | NoV GII.17[P17] | NoV GII | NoV GII.17[P17] | 100 |
| **Patient 12** | 1 | 10/02/2024 | neg | pos | neg | neg | NoV GII.17[P17] | NoV GII | NoV GII.17[P17] | 99.8 |
| **Patient 13** | 1 | 10/02/2024 | neg | neg | neg | neg | NoV GII.17[P17] | NoV GII | NoV GII.17[P17] | 99.4 |
| **Patient 14** | 1 | 10/02/2024 | neg | neg | neg | neg | NoV GII.17[P17] | NoV GII | NoV GII.17[P17]  Human rhinovirus 1 | 99.4  100 |
| **Patient 15** | 1 | 12/02/2024 | neg | neg | neg | neg | NoV GII.17[P17] | NoV GII | NoV GII.17[P17] | 100 |
| **Patient 16** | 1 | 15/02/2024 | neg | neg | neg | neg | neg | neg | NT | NA |
| **Patient 17** | 1 | 17/02/2024 | neg | neg | neg | neg | neg | neg | neg | NA |
| **Patient 18*** | 1 | 17/02/2024 | neg | neg | neg | neg | neg | NoV GII | NoV GII.17[P17] | 99.9 |
| **Patient 19*** | 1 | 17/02/2024 | neg | neg | neg | neg | NoV GII.17[P17] | NoV GII | NoV GII.17[P17] | 100 |
|  | 2 | 19/02/2024 | neg | neg | neg | neg | NoV GII.17[P17] | NoV GII | NoV GII.17[P17] | 100 |
| **Patient 20** | 1 | 18/02/2024 | neg | neg | neg | *C. diff* toxin B^‡^ | NoV GII.17[P17] | NoV GII  *C. diff* toxin A+B | NoV GII.17[P17] | 99.9 |
| **Patient 21** | 1 | 19/02/2024 | neg | neg | neg | neg | NoV GII.17[P17] | NoV GII | NoV GII.17[P17] | 99.9 |
| **Patient 22*** | 1 | 19/02/2024 | neg | neg | neg | neg | neg | neg | NT | NA |
|  | 2 | 22/02/2024 | neg | neg | neg | neg | neg | neg | NT | NA |
| **Patient 23*** | 1 | 20/02/2024 | neg | neg | neg | neg | NoV GII.17[P17] | NoV GII | NoV GII.17[P17] | 99.9 |
| **Patient 24*** | 1 | 20/02/2024 | neg | neg | neg | neg | neg | neg | NT | NA |
| **Patient 25*** | 1 | 21/02/2024 | neg | neg | neg | neg | neg | neg | NT | NA |
| **Patient 26** | 1 | 21/02/2024 | neg | neg | neg | neg | neg | neg | NT | NA |
| **Patient 27** | 1 | 22/02/2024 | neg | neg | neg | *C. diff* toxin B^‡^ | neg | *C. diff* toxin A+B | NT | NA |
| **Patient 28*** | 1 | 23/02/2024 | neg | neg | neg | neg | neg | neg | NT | NA |
|  | 2 | 29/02/2024 | neg | neg | neg | neg | neg | neg | NT | NA |
|  | 3 | 15/03/2024 | neg | neg | neg | neg | neg | neg | neg | NA |
| **Patient 29*** | 1 | 23/02/2024 | neg | neg | neg | neg | NoV GII.17[P17] | NoV GII | NoV GII.17[P17] | 100 |
| **Patient 30** | 1 | 25/02/2024 | neg | neg | neg | neg | neg | neg | NT | NA |
| **Patient 31** | 1 | 27/02/2024 | neg | neg | neg | neg | NoV GII.17[P17] | NoV GII | NoV GII.17 | 5.3 |
| **Patient 32*** | 1 | 28/02/2024 | neg | neg | neg | neg | neg | NoV GII  *Campylobacter spp*. | NT | NA |
| **Outbreak 2** | | | | | | | | | | |
| **Patient 1** | 1 | 06/05/2024 | neg | pos | neg | neg | NoV GII.17[P17] | NoV GII | NoV GII.17[P17] | 99.7 |
| **Patient 2** | 1 | 06/05/2024 | neg | neg | neg | neg | NoV GII.17[P17] | NoV GII | NT | NA |
| **Patient 3** | 1 | 06/05/2024 | neg | neg | neg | neg | neg | neg | NT | NA |
|  | 2 | 09/05/2024 | neg | pos | neg | neg | NoV GII.17[P17] | NoV GII | NoV GII.17[P17]  Human parainfluenza virus 3 | 100  99.6 |
|  | 3 | 25/05/2024 | neg | neg | neg | neg | NoV GII.17[P17] | NoV GII | NoV GII.17[P17] | 99.9 |
| **Patient 4** | 1 | 06/05/2024 | neg | neg | neg | neg | NoV GII.17[P17] | NoV GII | NT | NA |
| **Patient 5** | 1 | 06/05/2024 | neg | neg | neg | neg | NoV GII.17[P17] | NoV GII | neg | NA |
|  | 2 | 06/05/2024 | neg | neg | neg | neg | NoV GII.17[P17] | NoV GII  NoV GI  ETEC (lt/st) | NT | NA |
| **Patient 6** | 1 | 06/05/2024 | neg | neg | neg | neg | neg | neg | NT | NA |
|  | 2 | 07/05/2024 | neg | neg | neg | neg | neg | neg | NT | NA |
| **Patient 7** | 1 | 07/05/2024 | neg | pos | neg | neg | NoV GII.17[P17] | NoV GII | NT | NA |
| **Patient 8** | 1 | 06/05/2024 | neg | pos | neg | neg | neg | neg | NT | NA |
| **Patient 9** | 1 | 07/05/2024 | neg | pos | neg | neg | NoV GII.17[P17] | NoV GII | NoV GII.17[P17] | 99.9 |
| **Patient 10** | 1 | 07/05/2024 | neg | neg | neg | neg | NoV GII.17[P17] | NoV GII  ETEC (lt/st) | NT | NA |
|  | 2 | 07/05/2024 | neg | neg | neg | neg | NoV GII.17[P17] | NoV GII | NT | NA |
| **Patient 11** | 1 | 07/05/2024 | neg | neg | neg | neg | neg | neg | NT | NA |
|  | 2 | 10/05/2024 | neg | neg | neg | neg | neg | neg | NT | NA |
| **Patient 12** | 1 | 07/05/2024 | neg | neg | neg | *C. diff* toxin B^‡^ | neg | *C. diff* toxin A+B  *Salmonella* *spp.* | NT | NA |
| **Patient 13** | 1 | 07/05/2024 | neg | neg | neg | neg | neg | Invalid | NT | NA |
| **Patient 14** | 1 | 08/05/2024 | neg | neg | neg | neg | neg | NoV GII | NT | NA |
| **Patient 15** | 1 | 09/05/2024 | neg | neg | neg | neg | NoV GII.17[P17] | NoV GII | NoV GII.17[P17] | 100 |
| **Patient 16** | 1 | 10/05/2024 | neg | neg | neg | neg | neg | NoV GII | NT | NA |
| **Patient 17** | 1 | 10/05/2024 | neg | neg | neg | *C. diff* toxin B^‡^ | neg | *C. diff* toxin A+B | NT | NA |
| **Patient 18** | 1 | 11/05/2024 | neg | neg | neg | *C. diff* toxin B^‡^ | neg | *C. diff* toxin A+B | NT | NA |
| **Patient 19** | 1 | 12/05/2024 | neg | neg | neg | neg | neg | neg | NT | NA |
| **Patient 20** | 1 | 13/05/2024 | neg | neg | neg | neg | neg | neg | NT | NA |
| **Patient 21** | 1 | 12/05/2024 | neg | neg | neg | neg | neg | neg | NT | NA |
| **Patient 22** | 1 | 21/05/2024 | neg | neg | neg | neg | neg | Rotavirus | NoV GII.17[P17]  Rotavirus A | 30.3  95.1 |

*Patients admitted to Oncohematology; ^‡^PCR-method (GeneXpert – Cepheid) does not detect toxin A; *C. diff: Clostridium difficile;* ETEC (lt/st): Enterotoxigenic *Escherichia coli* (heat-labile/heat-stable); NoV: Norovirus; NT: not tested; NA: not applicable; neg: negative; pos: positive

**Supplementary Table 2.** Reason of hospitalization among laboratory-analyzed patients by outbreak and hospital unit.

|  |  | **Outbreak 1** | | **Outbreak 2** |
| --- | --- | --- | --- | --- |
| **Treatment** | **Overall** N = 54 | **Oncohematology** N = 9 | **Geriatrics** N = 23 | **Geriatrics**  N = 22 |
| Anemia | 2 (3.7) | 0 (0.0) | 2 (8.7) | 0 (0.0) |
| Cancer | 5 (9.3) | 2 (22.2) | 3 (13.0) | 3 (13.6) |
| Dyspnea | 3 (5.6) | 0 (0.0) | 0 (0.0) | 3 (13.6) |
| Functional deterioration | 2 (3.7) | 0 (0.0) | 2 (8.7) | 0 (0.0) |
| Gastrointestinal bleeding | 2 (3.7) | 0 (0.0) | 0 (0.0) | 2 (9.1) |
| Gastrointestinal symptoms | 3 (5.6) | 0 (0.0) | 0 (0.0) | 3 (13.6) |
| Infection (excl. pneumonia) | 6 (11.1) | 2 (22.2) | 1 (4.3) | 3 (13.6) |
| Injury | 3 (5.6) | 0 (0.0) | 0 (0.0) | 3 (13.6) |
| Neurological condition | 3 (5.6) | 1 (11.1) | 1 (4.3) | 1 (4.5) |
| Pneumonia | 16 (29.6) | 0 (0.0) | 9 (39.1) | 7 (31.8) |
| Stroke | 5 (9.3) | 4 (44.4) | 1 (4.3) | 0 (0.0) |
| Other | 3 (5.6) | 0 (0.0) | 3 (13.0) | 0 (0.0) |
| Unknown | 1 (1.9) | 0 (0.0) | 1 (4.3) | 0 (0.0) |

**Supplementary Table 3.** List of treatments laboratory-analyzed patients received during their hospitalization period by outbreak and hospital unit.

|  |  | **Outbreak 1** | | **Outbreak 2** |
| --- | --- | --- | --- | --- |
| **Treatment** | **Overall** N = 54 | **Oncohematology** N = 9 | **Geriatrics** N = 23 | **Geriatrics**  N = 22 |
| **Antibiotics** |  |  |  |  |
| Amoxicillin-clavulanate | 7 (13.0) | 0 (0.0) | 4 (17.4) | 3 (13.6) |
| Azithromycin | 4 (7.4) | 0 (0.0) | 3 (13.0) | 1 (4.5) |
| Cefepime | 1 (1.9) | 0 (0.0) | 1 (4.3) | 0 (0.0) |
| Cefixime | 2 (3.7) | 1 (11.1) | 1 (4.3) | 0 (0.0) |
| Ceftazidime | 1 (1.9) | 0 (0.0) | 1 (4.3) | 0 (0.0) |
| Ceftriaxone | 8 (14.8) | 0 (0.0) | 6 (26.1) | 2 (9.1) |
| Cloxacillin | 1 (1.9) | 0 (0.0) | 0 (0.0) | 1 (4.5) |
| Ertapenem | 1 (1.9) | 0 (0.0) | 1 (4.3) | 0 (0.0) |
| Levofloxacin | 2 (3.7) | 0 (0.0) | 2 (8.7) | 0 (0.0) |
| Linezolid | 1 (1.9) | 0 (0.0) | 1 (4.3) | 0 (0.0) |
| Meropenem | 5 (9.3) | 2 (22.2) | 2 (8.7) | 1 (4.5) |
| Nitrofurantoin | 1 (1.9) | 0 (0.0) | 0 (0.0) | 1 (4.5) |
| Piperacillin-Tazobactam | 2 (3.7) | 0 (0.0) | 2 (8.7) | 0 (0.0) |
| Vancomycin | 5 (9.3) | 2 (22.2) | 2 (8.7) | 1 (4.5) |
| **Antifungals** |  |  |  |  |
| Amphotericin B | 3 (5.6) | 3 (33.3) | 0 (0.0) | 0 (0.0) |
| Posaconazole | 2 (3.7) | 2 (22.2) | 0 (0.0) | 0 (0.0) |
| **Antivirals** |  |  |  |  |
| Remdesevir | 1 (1.9) | 0 (0.0) | 1 (4.3) | 0 (0.0) |
| **Antineoplastics** |  |  |  |  |
| Cytarabine | 2 (3.7) | 2 (22.2) | 0 (0.0) | 0 (0.0) |
| Idarubicin | 2 (3.7) | 2 (22.2) | 0 (0.0) | 0 (0.0) |
| Midostaurin | 1 (1.9) | 1 (11.1) | 0 (0.0) | 0 (0.0) |
| **Other treatments** |  |  |  |  |
| Antiaggregant | 1 (1.9) | 1 (11.1) | 0 (0.0) | 0 (0.0) |
| Corticosteroids | 1 (1.9) | 0 (0.0) | 1 (4.3) | 0 (0.0) |
| Enema | 2 (3.7) | 1 (11.1) | 1 (4.3) | 0 (0.0) |
| Furosemide | 1 (1.9) | 0 (0.0) | 1 (4.3) | 0 (0.0) |
| Laxatives | 2 (3.7) | 0 (0.0) | 1 (4.3) | 1 (4.5) |
| Movicol | 1 (1.9) | 0 (0.0) | 1 (4.3) | 0 (0.0) |
| Salbutamol | 1 (1.9) | 0 (0.0) | 1 (4.3) | 0 (0.0) |
| Others | 13 (24.1) | 0 (0.0) | 0 (0.0) | 13 (59.1) |

**Supplementary Table 4.** Comorbidities of all laboratory-analyzed patients by outbreak and hospital unit.

|  |  | **Outbreak 1** | | **Outbreak 2** |
| --- | --- | --- | --- | --- |
| **Comorbidity** | **Overall** N = 54 | **Oncohematology** N = 9 | **Geriatrics** N = 23 | **Geriatrics**  N = 22 |
| **Cancer** |  |  |  |  |
| Gastric cancer | 2 (3.7) | 0 (0.0) | 1 (4.3) | 1 (4.5) |
| Lung cancer | 1 (1.9) | 0 (0.0) | 0 (0.0) | 1 (4.5) |
| Metastatic colorectal cancer | 1 (1.9) | 0 (0.0) | 1 (4.3) | 0 (0.0) |
| Multiple myeloma | 1 (1.9) | 1 (11.1) | 0 (0.0) | 0 (0.0) |
| Prostate cancer | 1 (1.9) | 0 (0.0) | 1 (4.3) | 0 (0.0) |
| Urachal tumor | 1 (1.9) | 1 (11.1) | 0 (0.0) | 0 (0.0) |
| **Cardiovascular disease** |  |  |  |  |
| Atrial fibrillation | 13 (24.1) | 1 (11.1) | 7 (30.4) | 5 (22.7) |
| Heart disease | 5 (9.3) | 0 (0.0) | 5 (21.7) | 0 (0.0) |
| Heart failure | 3 (5.6) | 1 (11.1) | 1 (4.3) | 1 (4.5) |
| Hypertension | 41 (75.9) | 6 (66.7) | 19 (82.6) | 16 (72.7) |
| Hypertensive heart disease | 1 (1.9) | 0 (0.0) | 1 (4.3) | 0 (0.0) |
| Myocardial infarction | 1 (1.9) | 0 (0.0) | 1 (4.3) | 0 (0.0) |
| Pulmonary hypertension | 2 (3.7) | 1 (11.1) | 1 (4.3) | 0 (0.0) |
| **Endocrine and metabolic disorders** |  |  |  |  |
| Diabetes mellitus | 18 (33.3) | 3 (33.3) | 6 (26.1) | 9 (40.9) |
| Dyslipidemia | 24 (44.4) | 5 (55.6) | 7 (30.4) | 12 (54.5) |
| Hypothyroidism | 3 (5.6) | 0 (0.0) | 3 (13.0) | 0 (0.0) |
| Obesity | 3 (5.6) | 0 (0.0) | 1 (4.3) | 0 (0.0) |
| **Gastrointestinal disorders** |  |  |  |  |
| Constipation | 1 (1.9) | 0 (0.0) | 1 (4.3) | 0 (0.0) |
| Diverticulosis | 1 (1.9) | 0 (0.0) | 1 (4.3) | 0 (0.0) |
| Gastrectomy | 1 (1.9) | 0 (0.0) | 0 (0.0) | 1 (4.5) |
| Ulcerative colitis | 2 (3.7) | 1 (11.1) | 1 (4.3) | 0 (0.0) |
| **Genitourinary disorders** |  |  |  |  |
| Benign prostatic hyperplasia | 1 (1.9) | 0 (0.0) | 1 (4.3) | 0 (0.0) |
| Chronic kidney disease | 5 (9.3) | 1 (11.1) | 1 (4.3) | 3 (13.6) |
| **Neurological disorders** |  |  |  |  |
| Dementia | 1 (1.9) | 0 (0.0) | 1 (4.3) | 0 (0.0) |
| Stroke | 3 (5.6) | 0 (0.0) | 2 (8.7) | 1 (4.5) |
| **Other disorders** |  |  |  |  |
| Anemia | 2 (3.7) | 0 (0.0) | 2 (8.7) | 0 (0.0) |
| Polyarthritis | 1 (1.9) | 1 (11.1) | 0 (0.0) | 0 (0.0) |
| Sarcoidosis | 1 (1.9) | 0 (0.0) | 0 (0.0) | 1 (4.5) |
| Steatosis | 1 (1.9) | 0 (0.0) | 1 (4.3) | 0 (0.0) |
| **Respiratory disorders** |  |  |  |  |
| Asthma | 6 (11.1) | 0 (0.0) | 5 (21.7) | 0 (0.0) |
| Bronchitis | 1 (1.9) | 0 (0.0) | 1 (4.3) | 0 (0.0) |
| COPD | 6 (11.1) | 0 (0.0) | 1 (4.3) | 5 (22.7) |
| Obstructive sleep apnea  syndrome | 1 (1.9) | 0 (0.0) | 1 (4.3) | 0 (0.0) |
| Respiratory insufficiency | 1 (1.9) | 0 (0.0 | 1 (4.3) | 0 (0.0) |
| **Vascular ischemic disorders** |  |  |  |  |
| Intestinal ischemia | 1 (1.9) | 0 (0.0 | 1 (4.3) | 0 (0.0) |
| Lower limb ischemia | 1 (1.9) | 0 (0.0 | 1 (4.3) | 0 (0.0) |

**Supplementary Table 5.** Gelman-Rubin Convergence Diagnostics (A) and Effective Sample Sizes (B) for both outbreaks.

**A**

|  | **Outbreak 1** | | **Outbreak 2** | |
| --- | --- | --- | --- | --- |
| **Parameter** | **Point estimate** | **Upper CI** | **Point estimate** | **Upper CI** |
| **eps** | 1 | 1.01 | 1 | 1 |
| **lambda** | 1 | 1 | 1 | 1 |
| **like** | 1 | 1 | 1 | 1.01 |
| **mu** | 1 | 1 | 1 | 1 |
| **pi** | 1 | 1 | 1 | 1 |
| **post** | 1 | 1 | 1 | 1.01 |
| **prior** | 1 | 1 | 1 | 1 |

**B**

| **Parameter** | **Outbreak 1** | **Outbreak 2** |
| --- | --- | --- |
| **eps** | 3791.523 | 3463.554 |
| **lambda** | 3604.000 | 3682.660 |
| **like** | 3604.000 | 3604.000 |
| **mu** | 3653.879 | 3604.000 |
| **pi** | 3604.000 | 3246.766 |
| **post** | 3655.526 | 3604.000 |
| **prior** | 3604.000 | 3251.849 |

**
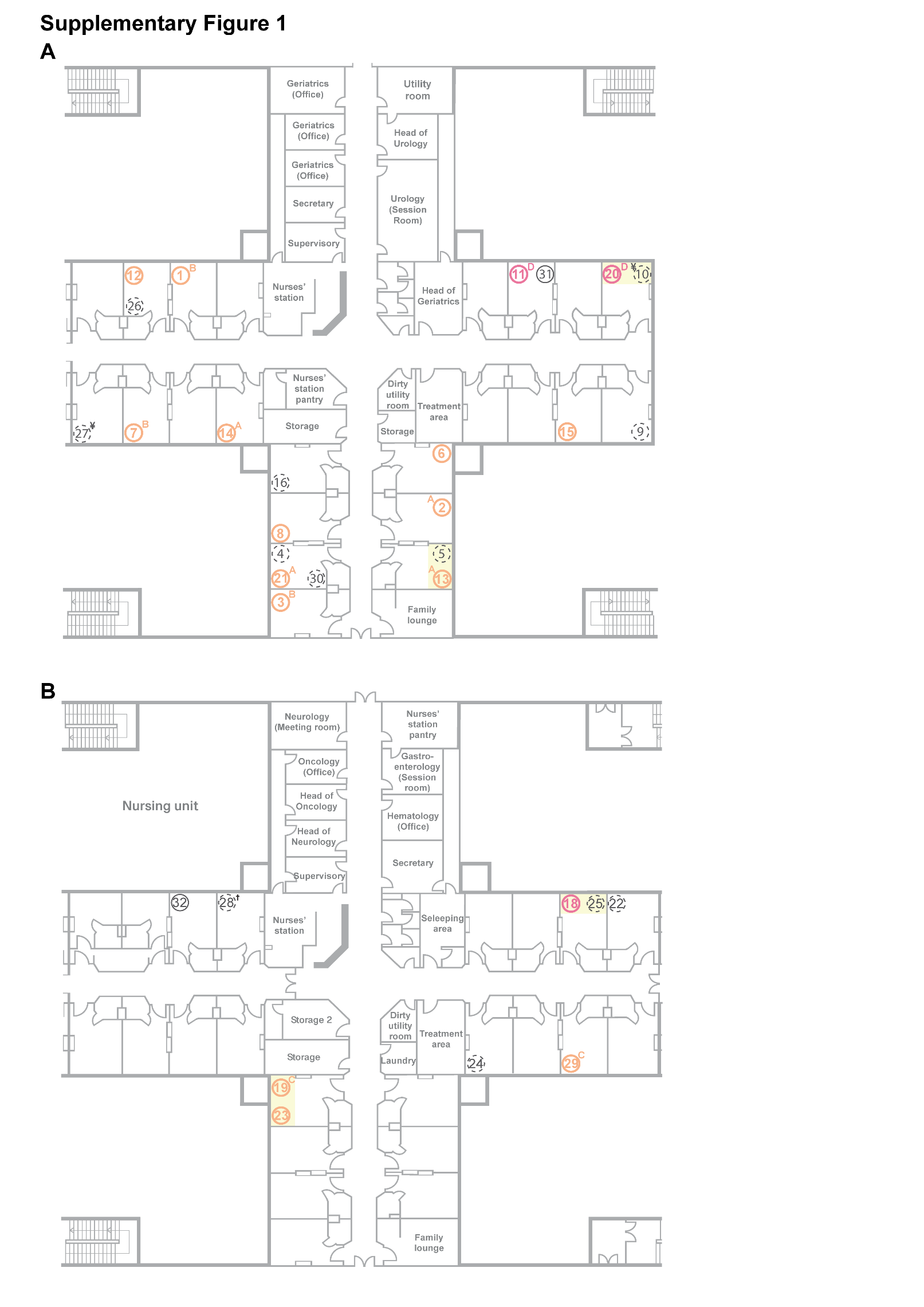
**

**
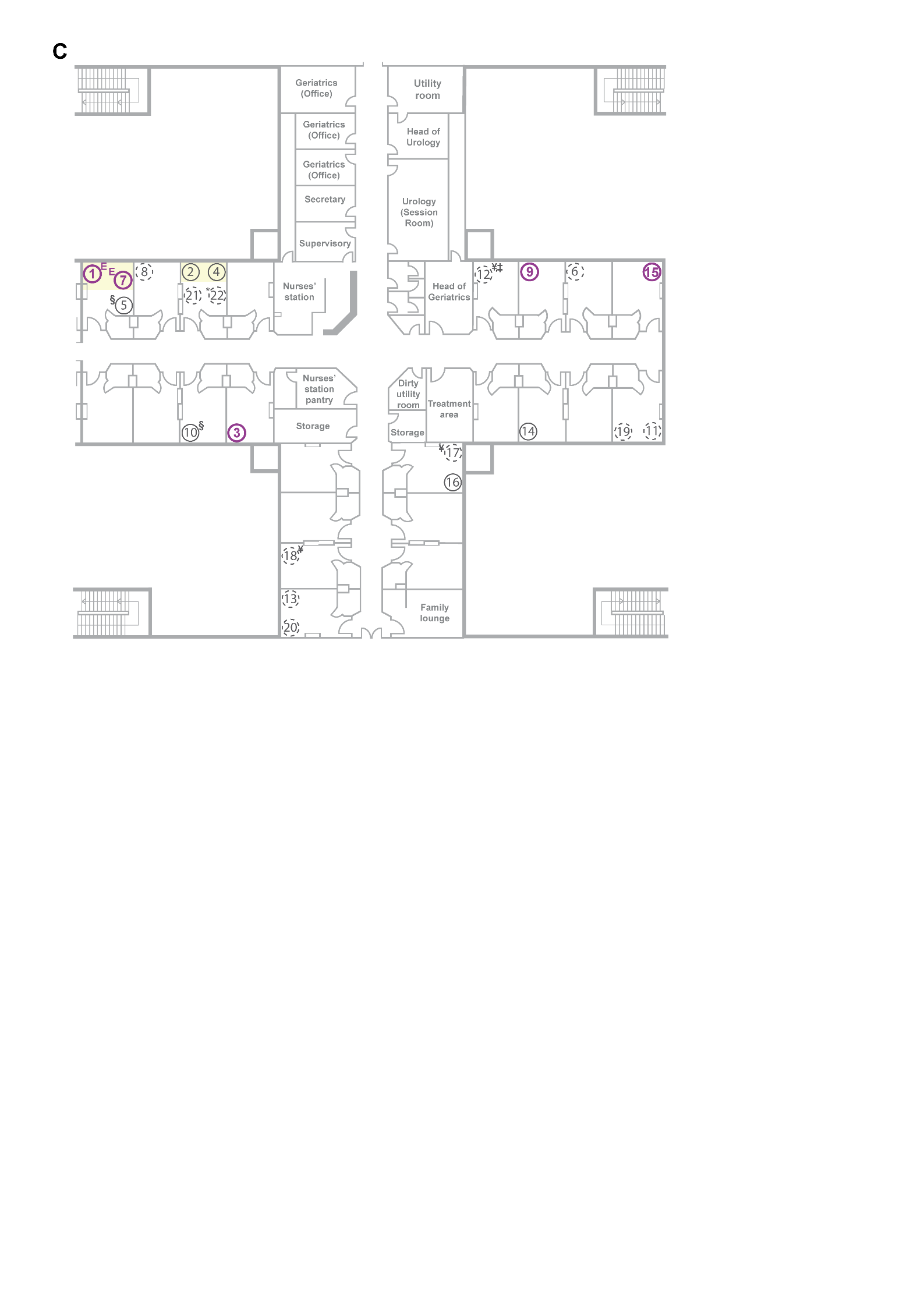
**

**Supplementary Figure 1.** Layouts of hospital wards with room allocation for symptomatic patients. **A**. Geriatrics, Outbreak-1; **B**. Oncohematology, Outbreak-1; **C**. Geriatrics, Outbreak-2. Numbers inside circles indicate Patient IDs. Dashed circles represent symptomatic patients; solid circles indicate NoV-positive patients confirmed by LiquidArray^®^. Bold circles denote patients from whom whole-genome NoV sequences were obtained. Letters next to circles identify patients belonging to groups with identical sequences. Symbols indicate infection with other enteropathogens detected by LiquidArray®): ^¥^*C. difficile* tox A+B; ^§^ETEC (lt/st); ^‡^*Salmonella* spp. IND; ^†^*Campylobacter* spp.; *Rotavirus. Light yellow rectangles mark overlapping hospitalization period in the same room.

**
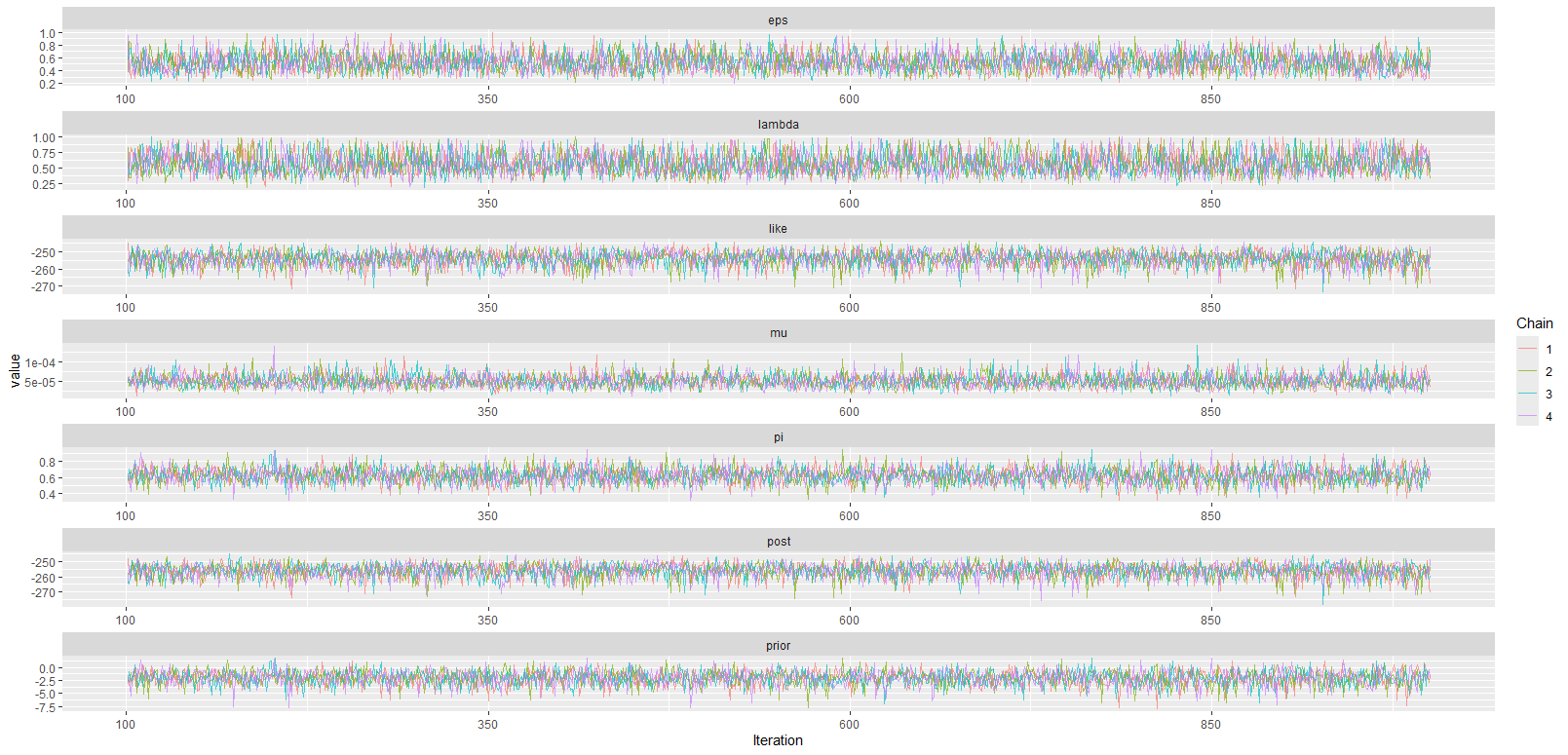
**

**A**

**
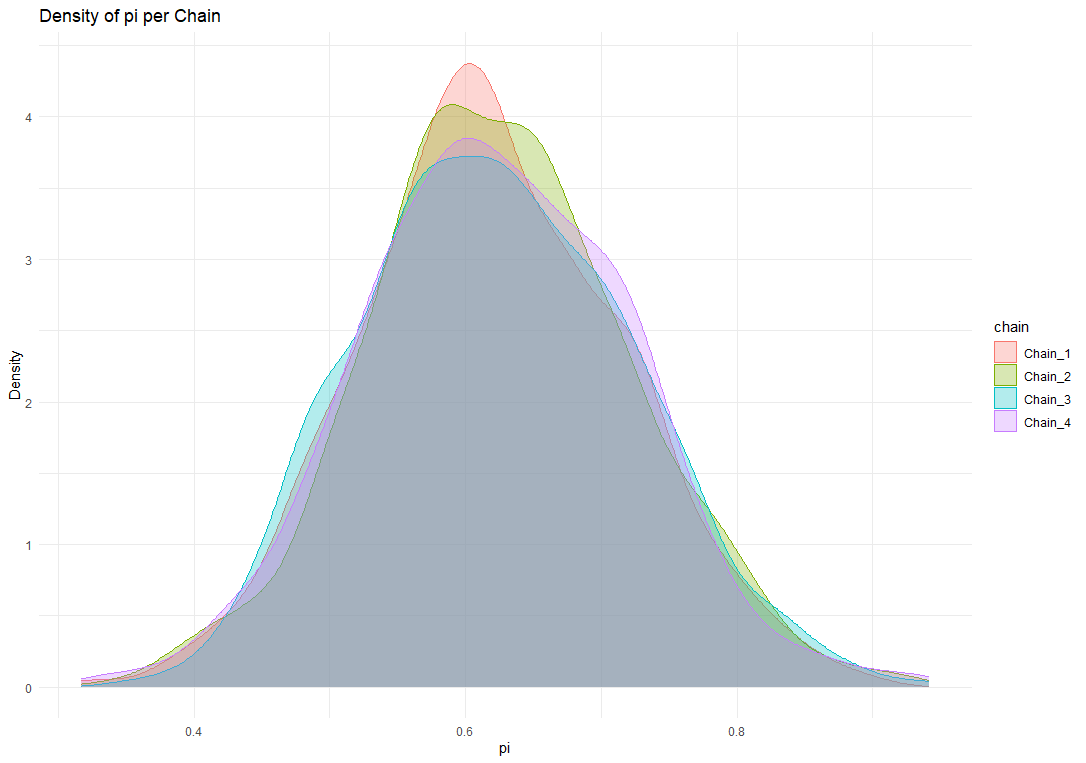
**

**C**

**B**

**
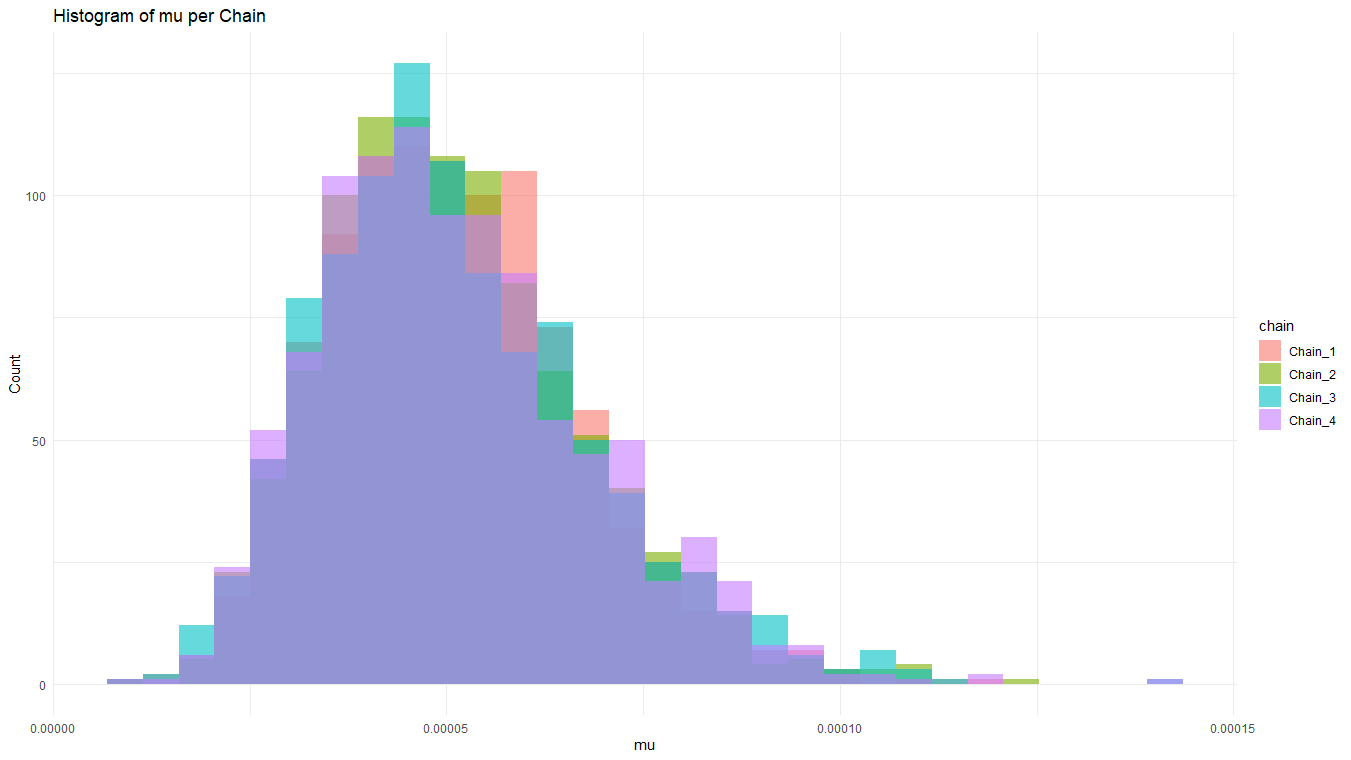
**

**
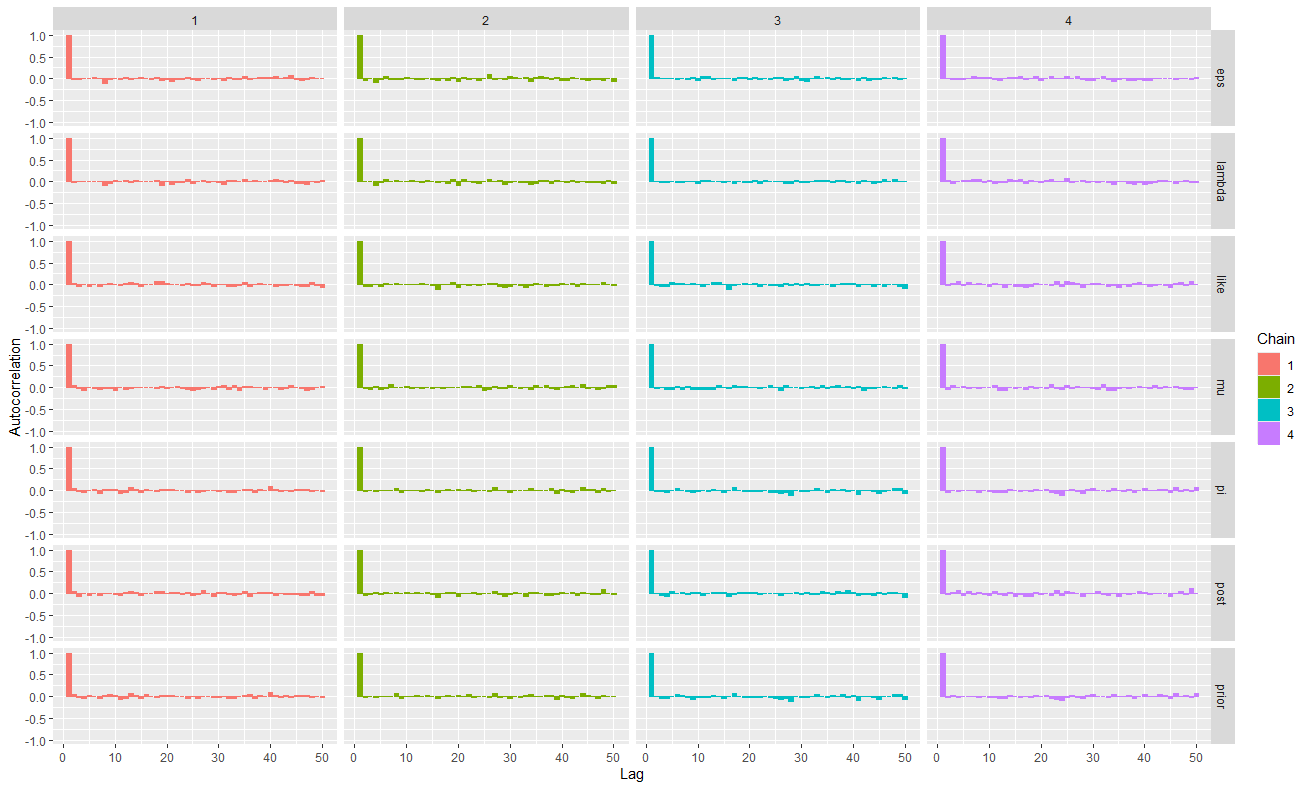
**

**D**

**Supplementary Figure 2** MCMC convergence diagnostics for *outbreaker2* models for Outbreak-1. **A.** Traceplots; **B.** Density plot of pi; **C.** Histogram of mu; **D.** Autocorrelation of relevant parameters.

**A**

**
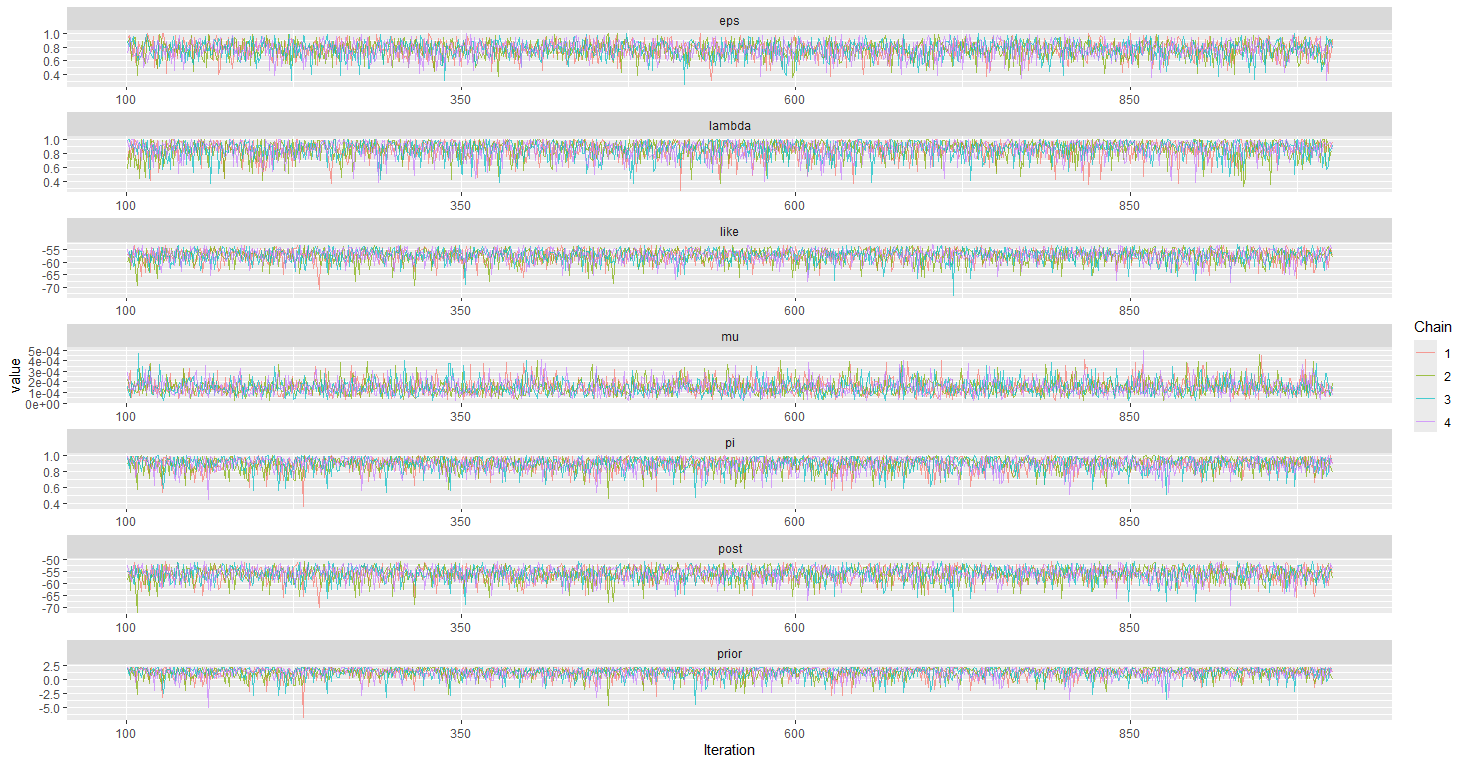
**

**
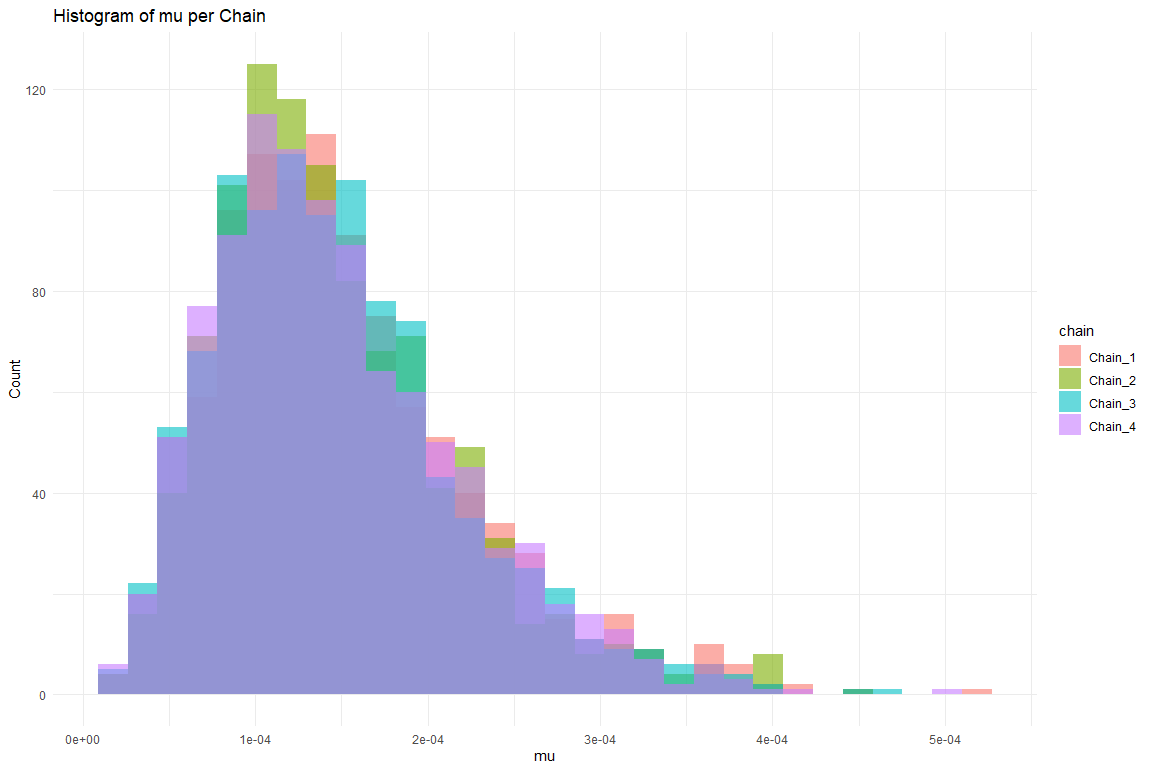

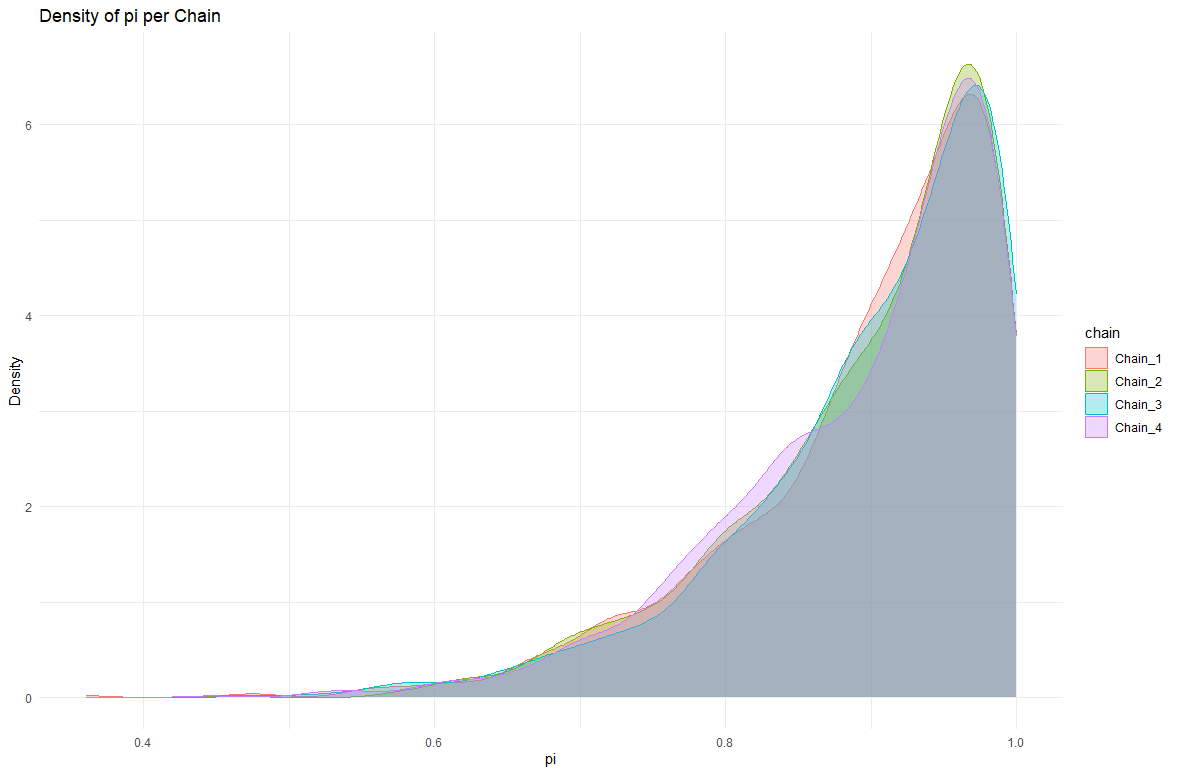
**

**C**

**B**

**
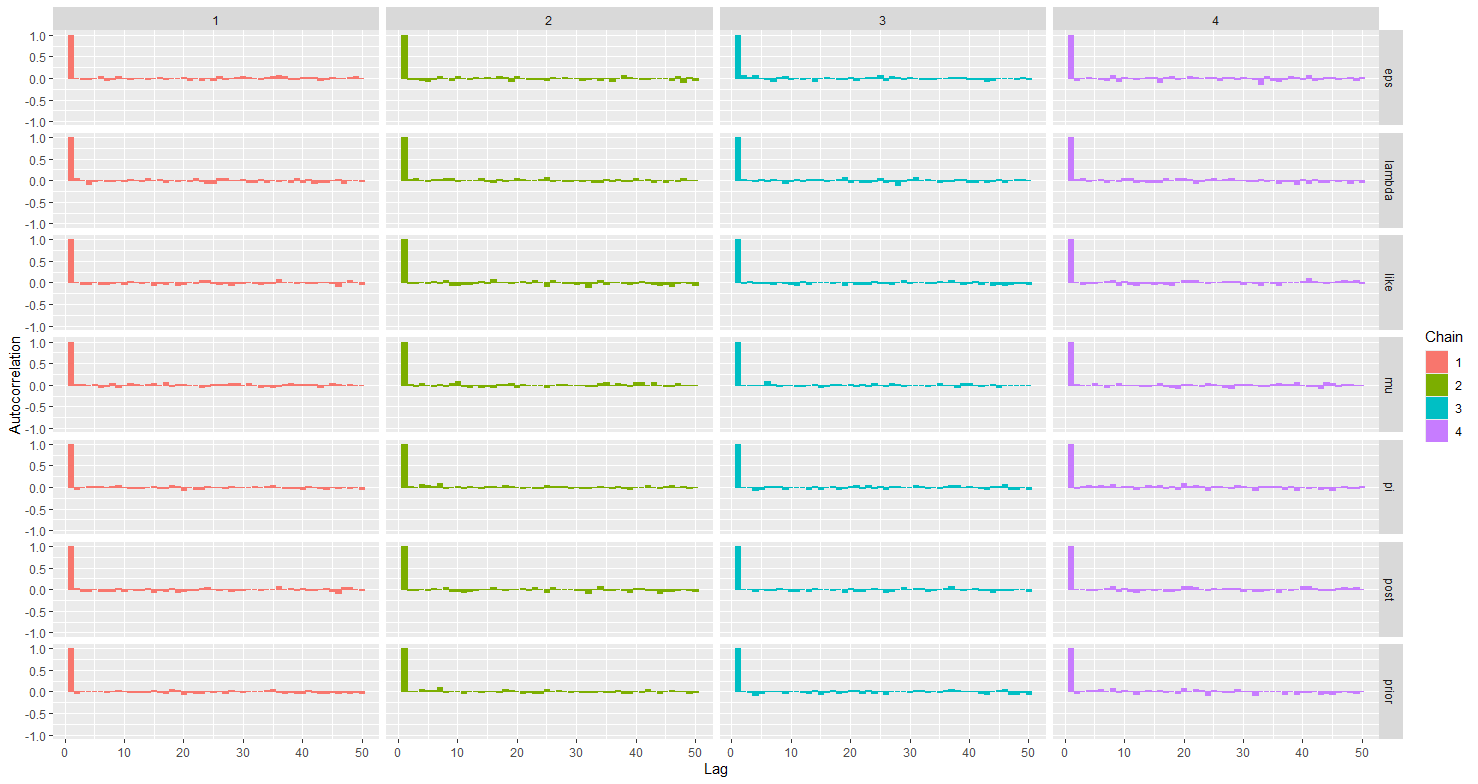
**

**D**

**Supplementary Figure 3** MCMC convergence diagnostics for *outbreaker2* models for Outbreak-2. **A.** Traceplots; **B.** Density plot of pi; **C.** Histogram of mu; **D.** Autocorrelation of relevant parameters.
